# Hybrid pH Responsive Supramolecular Polymers Through the Combination of the Ureido–Pyrimidinone Motif with β‐Sheet Peptide Sequences

**DOI:** 10.1002/chem.202500429

**Published:** 2025-05-15

**Authors:** Riccardo Bellan, David Attia, Marcel H. P. van Genderen, Patricia Y. W. Dankers

**Affiliations:** ^1^ Institute for Complex Molecular Systems and Department of Biomedical Engineering Eindhoven University of Technology 5600 MB Eindhoven The Netherlands; ^2^ Department of Chemical Engineering and Chemistry Eindhoven University of Technology 5600 MB Eindhoven The Netherlands

**Keywords:** assemblies, hydrogen bonding unit, peptides, supramolecular chemistry

## Abstract

Supramolecular biomaterials based on 1D supramolecular polymers in water replicate the fibrous and dynamic structures of natural architectures. Peptides, valued for their biocompatibility, are commonly employed as building blocks for supramolecular biomaterials, often in combination with large aromatic or hydrogen‐bonding groups at the *N*‐terminus to improve their structural stability. Herein, the self‐assembly properties of two β‐sheet peptides combined with the ureido–pyrimidinone self‐dimerizing motif in aqueous solution are investigated in detail. The assembly of the resulting molecules is demonstrated to be intimately dependent on the β‐sheet sequence, while the incorporation of a hydrophobic spacer enhances the assembly of the monomers, irrespective of the peptide sequence employed. Furthermore, the assembly of each monomer is significantly enhanced at pH = 3.0 while being stable between pH = 5.0 and 9.0. Only at pH = 12, upon enolate formation, the transition to random coil conformation is observed for all the monomers. The supramolecular polymers developed hereby point to fundamental design principle toward the development of UPy‐peptide based materials with tuneable properties and potential applications in the biomedical fields of research.

## Graphical abstract

The combination of the ureido–pyrimidinone motif with two different β‐sheet peptide sequences yielded supramolecular structures with significantly different morphologies in water. The resulting supramolecular nanostructures showed structural stability between pH = 5 and 9, while precipitation occurred at pH = 3.0 and random coil formation at pH 12. This work poses the basis for the development of novel responsive supramolecular polymers.

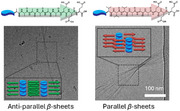

## 1 Introduction

Supramolecular polymers originate from the spontaneous organization of molecular building blocks, into more complex hierarchical structures via noncovalent interactions.^[^ ^1^, ^2^ ^]^ Naturally occurring supramolecular polymers, such as the cytoskeleton filaments,^[^ ^3^, ^4^ ^]^ collagen,^[^ ^5^ ^]^ and amyloid fibrils,^[^ ^6^ ^]^ are essential for living systems. They regulate many key functions including cell motility and cell division, as a result of the dynamics provided by the noncovalent interactions holding them.^[^ ^7^, ^8^ ^]^ Owing to their dynamic properties, natural supramolecular polymers have inspired scientists to develop robust yet dynamic supramolecular materials using natural building blocks.^[^ ^2^ ^]^ Among them, peptides gained particular interest due to their stability, synthetic feasibility, and sequence diversity.^[^ ^9^ ^]^ Furthermore, their intrinsic biocompatibility and biodegradability makes them attractive biomaterials.^[^ ^10^, ^11^ ^]^ Peptides can assemble into α‐helices or β‐sheets through hydrogen bonding depending on their amino acid sequence.^[^ ^12^, ^13^ ^]^ Short peptides that self‐assemble into nanofibers have been shown to efficiently perform as drug delivery systems.^[^ ^14^, ^15^ ^]^ Another class of self‐assembly peptides is represented by peptide amphiphiles (PA)s, where β‐sheet peptide sequences in combination with a lipidic tail at the *N*‐terminus can assemble into 1D structures through a combination of hydrophobic interactions and hydrogen bonding. Moreover, a polar head group is installed at the *C*‐terminus of PAs, usually composed of charged amino‐acid residues, to ensure the water solubility of the assembled structures.^[^ ^11^, ^16^ ^]^ Owing to their morphological features and tuneable dynamics, PAs have shown many applications as biomaterials to induce spinal cord and bone regeneration,^[^ ^17^, ^18^ ^]^ or as nanoplatforms for growth factors release.^[^ ^19^, ^20^ ^]^

On the other hand, large planar aromatic residues, such as naphthalene (Nap),^[^ ^21^ ^]^ phenothiazine (Ptz),^[^ ^22^ ^]^ carboxybenzyl,^[^ ^23^ ^]^ and 9‐fluorenylmethoxycarbonyl (Fmoc)^[^ ^24^ ^]^ groups, are conjugated to short β‐sheet peptide sequences at the *N*‐terminus to improve the structural integrity of the assemblies through π–π stacking. These systems can then be formulated into hydrogels as drug delivery platforms,^[^ ^25^, ^26^ ^]^ antimicrobial biomaterials,^[^ ^27^, ^28^ ^]^ as well as 3D scaffolds for tissue engineering.^[^ ^29^, ^30^ ^]^ Hydrogen bonding motifs have been combined with short peptide sequences with the same aims.^[^ ^31^, ^32^, ^33^, ^34^ ^]^ Benzene‐1,3,5‐tricarboxamide (BTA) molecules are known to form 1D fibers in water through a cooperative mechanism involving the combination of hydrophobic interactions, π–π stacking and hydrogen bonds between the amides.^[^ ^35^, ^36^ ^]^ BTA molecules have been conjugated to peptides mostly for improving the water solubility of the supramolecular polymers,^[^ ^37^, ^38^ ^]^ to introduce chirality^[^ ^39^ ^]^ and to easily introduce functionalities within the supramolecular structures.^[^ ^40^ ^]^ In comparison, molecules bearing the ureido–pyrimidinone (UPy) motif can dimerize through a self‐complementary four‐fold hydrogen bonding array,^[^ ^41^ ^]^ leading to the formation of flat dimeric surfaces that can stack through π–π stacking and result in 1D fibers in water via additional lateral hydrogen bonding, provided by urea groups.^[^ ^42^, ^43^ ^]^ Unlike BTAs, where the peptide sequence plays a structural role in the assembly of the resulting monomers, peptides have been conjugated to UPy molecules mostly at the periphery to serve as bioactive cues to be incorporated in host supramolecular polymers for developing platforms for growth factor release,^[^ ^44^, ^45^ ^]^ antimicrobial additives^[^ ^32^ ^]^ and to enhance cellular spreading and adhesion in artificial 3D networks.^[^ ^46^, ^47^ ^]^

Herein, two β‐sheet sequences are employed in combination with the UPy motif, to elucidate their structural role in the self‐assembly of the resulting supramolecular monomers in water. To this end, four hybrid UPy‐peptide derivatives are synthesized, featuring the A_6_E_3_ (**P1**) and V_3_A_3_E_3_ (**P2**) β‐sheet sequences in combination with the UPy motif, with or without an alkyl spacer. The findings of this work reveal that the choice of the peptide sequence affects both the UPy dimerization and the morphology of the aggregates in solution, while the incorporation of the alkyl spacer enhances both the UPy dimerization and the self‐assembly behavior of the monomers, regardless of the peptide sequence employed. Furthermore, given the pH responsiveness of both the UPy unit and the selected peptide sequences,^[^ ^18^, ^48^, ^49^, ^50^, ^51^ ^]^ the effect of pH on the assembly of each monomer is evaluated across a broad pH range, underlying the crucial role of the UPy dimerization in the supramolecular polymerization of the hybrid monomers. The findings of this work lay the foundation for the rational design of future responsive supramolecular materials with tuneable assembly properties.

## 2 Results and Discussion

**Figure 1:**
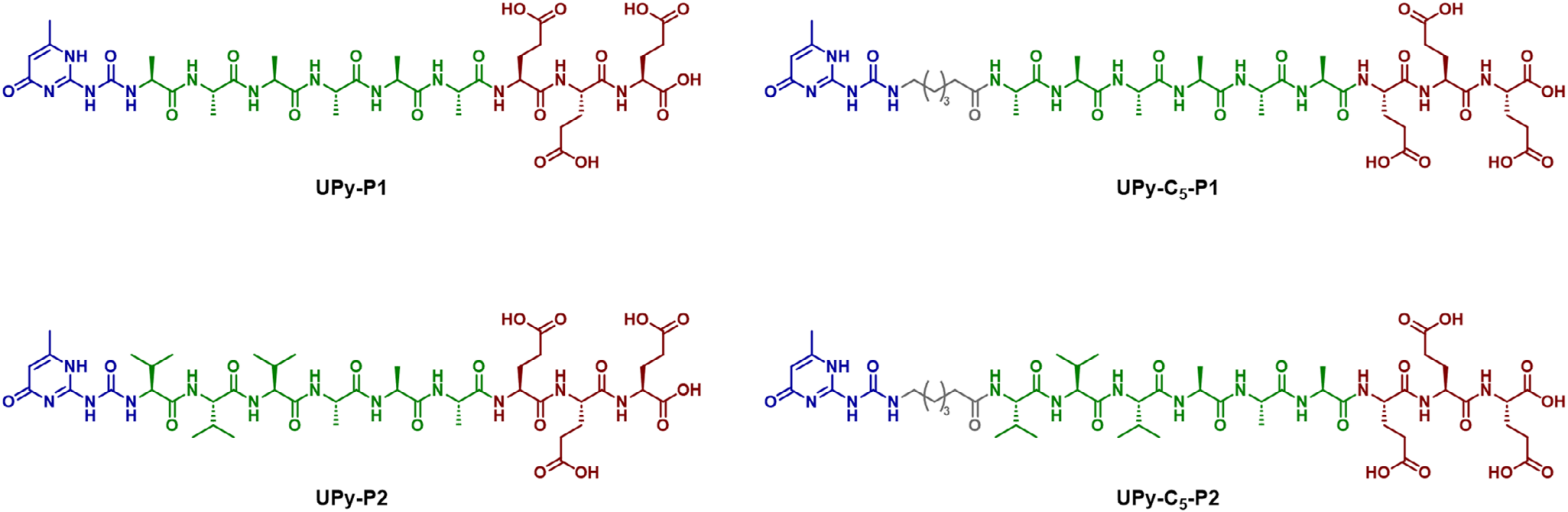
Chemical structure of the synthesized molecules.

The β‐sheet peptide sequences **P1** and **P2** are well‐known to form weak and strong β‐sheets, respectively.^[^ ^16^, ^18^, ^52^ ^]^ They consist of the hydrogen bonding sequences (green) A_6_ and V_3_A_3_ conjugated at the *C*‐terminus to the ionizable hydrophilic E_3_ sequence (red) to improve their water solubility. In this study, **P1** and **P2** are covalently conjugated at the *N*‐terminus to the hydrogen bonding UPy motif (blue) directly in **UPy‐P1** and **UPy‐P2** (Figure 1) or through a C_5_ spacer (grey) in between in **UPy‐C_5_‐P1** and **UPy‐C_5_‐P2** (Figure 1). The synthesis of the hybrid monomers is performed using standard fluorenylmethyloxycarbonyl (Fmoc) solid‐phase peptide synthesis^[^ ^53^ ^]^ on a glutamic acid preloaded Wang resin, followed by direct coupling of **UPy‐CDI** at the *N*‐terminus for **UPy‐P1** and **UPy‐P2** and of Fmoc‐6‐aminohexanoic acid for **UPy‐C_5_‐P1** and **UPy‐C_5_‐P2,** as reported in the supporting information (Scheme S2). Next, **UPy‐CDI** is coupled to the *N*‐terminus of the alkylated peptide sequences after Fmoc deprotection to provide **UPy‐C_5_‐P1** and **UPy‐C_5_‐P2** on the resin (Scheme S2). The final molecules are then removed from the solid support using a trifluoroacetic acid (TFA) based cleavage cocktail and purified by high‐performance liquid chromatography (HPLC) followed by freeze‐drying. The pure materials are finally characterized through liquid chromatography mass spectroscopy (LC‐MS) (Figure S1–S4).

**Figure 2:**
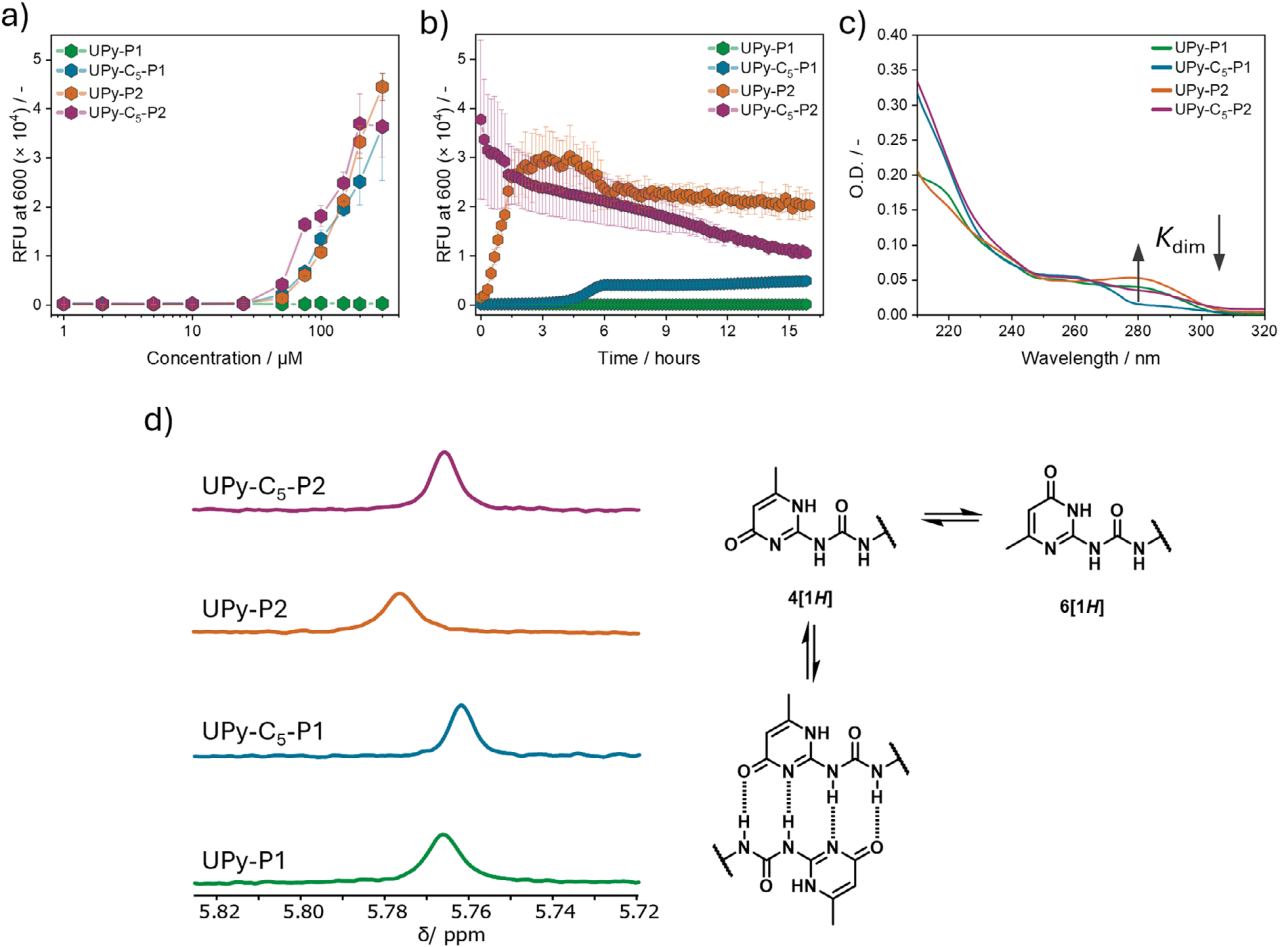
Self‐assembly analyses of the synthesized molecules in water at neutral pH. (a) Proteostat aggregation assay for the determination of the cac of each monomer (*C* = 1.0 µM to 300 µM) and for (b) assembly kinetic analysis (*C* = 100 µM). Data are presented as ± SD, *n* = 4. (c) UV–vis spectra of each monomers (*C* = 100 µM, T = 20 °C, *l* = 1.0 mm). (d) Partial stacked ^1^H‐NMR spectra of the assembled monomers in D_2_O (*C* = 500 µM) focusing on the chemical shift of the alkylidene proton of the UPy ring.

The self‐assembly behavior of the synthesized molecules in neutral water is firstly assessed via the Proteostat assay (Figure 2a), employed for probing amyloid‐like peptide nanostructures rich in *β*‐sheets.^[^ ^54^, ^55^, ^56^, ^57^ ^]^ The critical aggregation concentration (cac) is estimated to be 50 µM for **UPy‐P2**, 25 µM for both **UPy‐C_5_‐P1** and **UPy‐C_5_‐P2** while for **UPy‐P1** it was not determined in the explored concentration range (C = 1.0 µM–300 µM) owing to the lack of fluorescence increase. These results indicate that **UPy‐P1** is less prone to form *β*‐sheets than **UPy‐P2** as a result of the more polar peptide sequence, while the C_5_ spacer enhances the assembly of the monomers in water regardless of the peptide sequence used. ^1^H‐NMR spectroscopy in D_2_O (Figures S5–S9) shows similar percentage of 55 mol % of mobile UPy moiety and 49 mol % of *β*‐sheet sequence for **UPy‐P1** at room temperature, indicating that the remaining percentage of both molecular moieties present similar rigidity due to assembly formation.^[^ ^58^, ^59^, ^60^, ^61^ ^]^ The introduction of the C_5_ spacer in **UPy‐C_5_‐P1** decreases further the percentage of mobile UPy moiety and *β*‐sheet sequence similarly (34 mol %), indicating a lower degree of mobility of the more hydrophobic monomer compared to **UPy‐P1**. Conversely, the percentage of visible UPy moiety for both **UPy‐P2** and **UPy‐C_5_‐P2** is equal to 29 mol % and 22 mol %, respectively, indicating a slight decrease in mobility of the UPy ring provided by the alkyl spacer.

Similarly, the percentage of mobile *β*‐sheet sequence is 26 mol% for **UPy‐P2** and 22 mol% for **UPy‐C_5_‐P2**, indicating a decreased mobility of the peptide sequence for the monomer featuring the alkyl spacer. Irrespective of the monomer design, the percentage of mobile glutamic acids remains 100 mol% for each monomer, indicating full mobility of the hydrophilic groups. These results suggest that both the UPy moiety and the peptide sequences are located in the interior of the assemblies, while the ionized glutamic acids of each sequence at the water interface.

The assembly kinetic experiment performed above the cac of each monomer reveals differences based on the monomer design. By using the Proteostat assay (Figure 2b), **UPy‐P1** hardly shows any *β*‐sheet formation overtime, while the more hydrophobic **UPy‐C_5_‐P1** shows complete aggregation after 6 hours of incubation at room temperature. On the other hand, **UPy‐P2** and **UPy‐C_5_‐P2** exhibit faster assembly kinetics, with complete aggregation of the former after 3 hours and instant aggregation for the latter. These results indicate that the peptide sequence itself has an impact on the assembly kinetics of each monomer as well as the presence of the alkyl spacer for both the peptide sequences. UV–vis spectroscopy (Figure 2c) on the assembled monomers in water shows differences in absorption intensity at 280 nm, which corresponds to the non‐hydrogen bonded 6[1*H*] UPy tautomer, as previously described.^[^ ^62^, ^63^, ^64^ ^]^ Indeed, **UPy‐P1** displays greater absorbance than **UPy‐C_5_‐P1** at 280 nm, indicating that the alkyl spacer promotes the stabilization of the 4[1*H*] tautomer and its dimerization, while the UPy unit itself coupled to the **P1** sequence leads to a higher fraction of non‐dimerizing 6[1*H*] tautomer in solution. A similar trend is observed for **UPy‐P2** and **UPy‐C_5_‐P2**, confirming the importance of the hydrophobic spacer in stabilizing the 4[1*H*] tautomer rather than the 6[1*H*] one. However, by comparing **P1** and **P2**, it is clear that **P1** promotes the formation of 4[1*H*] tautomer more efficiently than **P2**, as demonstrated by the greater absorbance of the molecules bearing the latter at 280 nm. ^1^H‐NMR spectroscopy (Figure 2d) in D_2_O confirms further the aforementioned statement by showing a more upfield shifted signal of the alkylidene proton (5.75–5.79 ppm) on the UPy ring for **UPy‐P1** and **UPy‐C_5_‐P1** than **UPy‐P2** and **UPy‐C_5_‐P2**, indicating enhanced stacking of the UPy dimers for the former molecules.^[^ ^42^ ^]^ The decreased dimerization and stacking of the UPy observed for the monomers featuring the **P2** sequence might be due to multiple factors, such as the higher steric hindrance provided by the side chains and a different alignment of the β‐sheets formed by the respective monomers (vide infra*)*, which may hamper the dimerization and the stacking of the UPy rings.

The secondary structure of the assemblies formed by the synthesized monomers is elucidated through circular dichroism (CD) and Fourier‐transform infrared (FT‐IR) spectroscopy.

**Figure 3:**
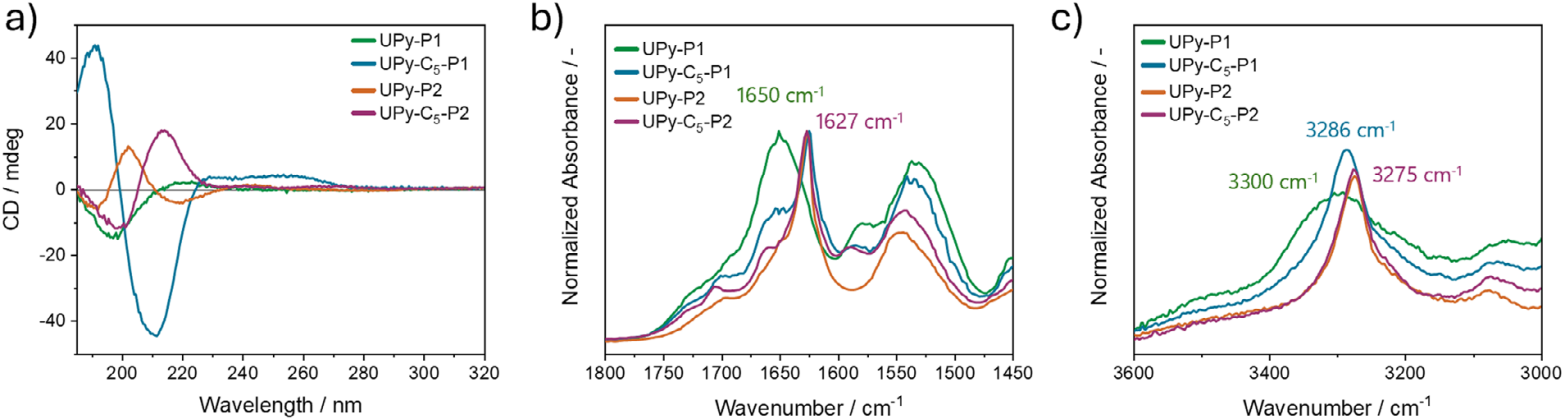
(a) CD spectra of the assembled monomers in MQ water (*C* = 100 µM, *T* = 20 °C, *l* = 1.0 mm) and (b and c) FT‐IR of each monomers in the solid state at 20 °C. (b) Zoom on the amide I and amide II region and (c) zoom of the amide A region.

In agreement with the results obtained from the Proteostat assay, the CD spectra of the different molecules in water show the formation of β‐sheets for all the monomers except for **UPy‐P1** (Figure 3a). Indeed, the CD spectrum of the latter presents a negative and a positive Cotton effect at 195 nm and at 220 nm, respectively, suggesting the presence of unorganized structures in solution resembling the random coil conformation.^[^ ^65^ ^]^ In contrast, the CD spectrum of **UPy‐C_5_‐P1** shows a positive and negative Cotton effect at 191 nm and 210 nm, respectively, pointing to the formation of anti‐parallel β‐sheets in solution.^[^ ^66^, ^67^ ^]^ The CD spectra of **UPy‐P2** and **UPy‐C_5_‐P2** are red‐shifted relative to the aforementioned one, with a maximum peak at 202 nm and 213 nm, respectively, suggesting the formation of parallel β‐sheets.^[^ ^16^, ^66^, ^68^ ^]^ Such redshift suggests an increased twisting of the nanostructures in solution for the monomer featuring the **P2** sequence.^[^ ^16^ ^]^ This effect is further amplified by the presence of the alkyl spacer, as evidenced by the additional redshift observed in the CD spectrum of **UPy‐C_5_‐P2** compared to **UPy‐P2**. Interestingly, the CD spectra of all the molecules maintain the same shape from 50 µM to 300 µM, indicating that the concentration does not affect the secondary structure of the assemblies regardless of the nature of the assembled species within the investigated range (Figure S10).

**Figure 4:**
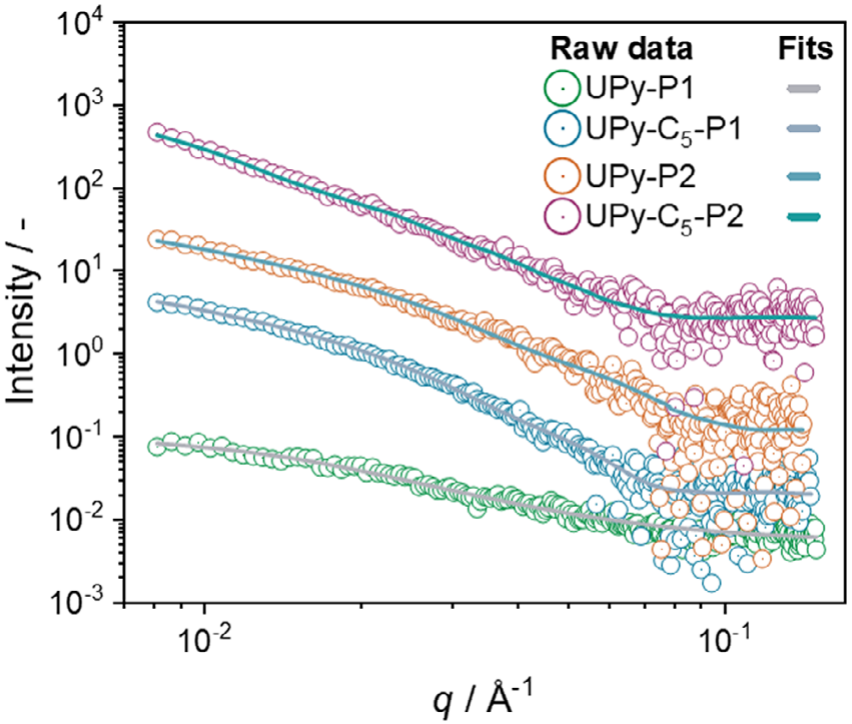
1D SAXS patterns (circles) and relative fits (solid lines) obtained from the assembled monomers in water (*C* = 0.5 mM). The patterns are shifted vertically for clarity.

FT‐IR measurements in bulk (Figure 3b,c) corroborates the CD results. Indeed, the shift observed from 1650 cm^−1^ for **UPy‐P1** to 1627 cm^−1^ for the rest of the molecules in the amide I region (Figure 3b) indicates β‐sheets formation for all the monomers except for **UPy‐P1**.^[^ ^50^, ^69^, ^70^ ^]^ Furthermore, the absorption bands at 3300 cm^−1^ and 3286 cm^−1^ in the amide A region (Figure 3c) for **UPy‐P1** and **UPy‐C_5_‐P1**, respectively, confirm the presence of more free NH groups for the former molecule, while **UPy‐P2** and **UPy‐C_5_‐P2** present the same vibration band at 3275 cm^−1^ which indicates the involvement of the N─H groups in hydrogen bonding formation.^[^ ^71^ ^]^ These results indicate that the **P2** sequence promotes the formation of stronger β‐sheets than **P1**, as previously described,^[^ ^18^ ^]^ and that the presence of the alkyl spacer enhances hydrogen bonding for both the peptide sequences. In agreement with the other results, cryoTEM does not show visible assemblies for **UPy‐P1** (Figure 6b), confirming the formation of more disorganized structures, while µm‐long twisted nanofibers are observed for **UPy‐C_5_‐P1** (Figure 6e). On the other hand, **UPy‐P2** (Figure 6h) and **UPy‐C_5_‐P2** (Figure 6k) form assemblies with similar morphology and larger diameter than those formed by **UPy‐C_5_‐P1**. Remarkably, the morphologies found for **UPy‐C_5_‐P1**, **UPy‐P2**, and **UPy‐C_5_‐P2** are consistent with the literature regarding the same peptide sequences.^[^ ^51^, ^67^ ^]^ The small‐angle X‐ray scattering (SAXS) patterns obtained from the assemblies formed by **UPy‐P1**, **UPy‐C_5_‐P1**, **UPy‐P2,** and **UPy‐C_5_‐P2** (Figure 4) in neutral water are consistent with previous CD and cryoTEM measurements.

The SAXS curve of **UPy‐P1** could be fitted best to the Debye gaussian coil model (Equation S1) (Table S1), confirming the formation of random coils with an average radius of gyration (*R_g_*) of 10.7 ± 0.3 nm. Conversely, the SAXS patterns of **UPy‐C_5_‐P1**, **UPy‐P2** and **UPy‐C_5_‐P2** could be fitted best to the parallelepiped model (Equation S2) (Table S1) where the fibers are assumed to be rectangular prism‐shaped, with A and B as the width and thickness, and C as the length of the fiber. The fittings indicate that while the cross‐section dimensions of **UPy‐C_5_‐P1** and **UPy‐P2** are similar, where the value of B spans from 16 nm to 18 nm, the cross‐section of **UPy‐C_5_‐P2** is more than two times larger and B ∼ 43 nm. These findings confirm that the alkyl spacer enhances the assembly and promotes the lateral aggregation of the supramolecular polymers formed by the respective monomers in solution.

**Figure 5:**
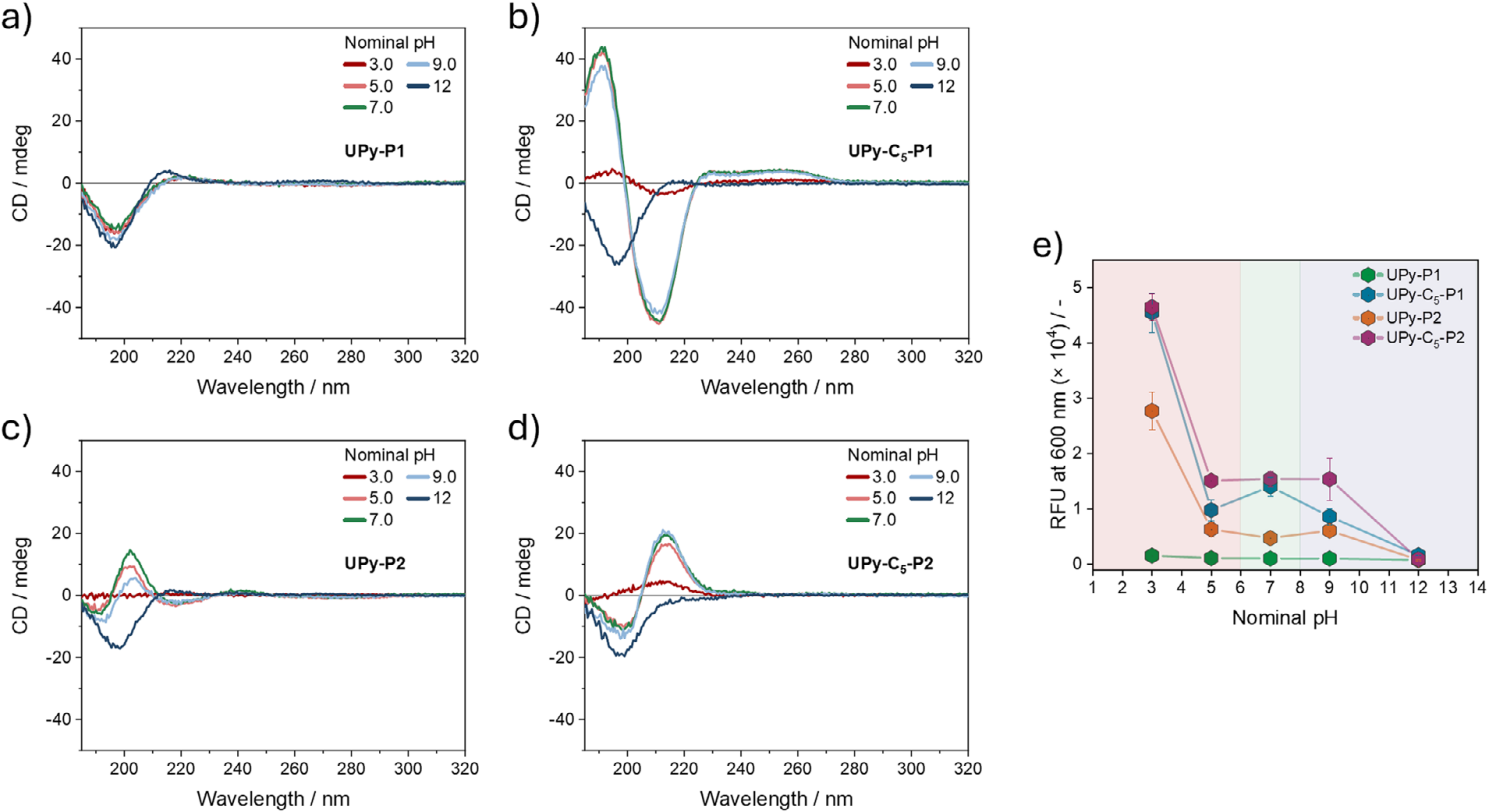
pH dependent CD spectra of (a) UPy‐P1, (b) UPy‐C_5_‐P1, (c) UPy‐P2, and (d) UPy‐C_5_‐P2 in water at a nominal pH of 3.0, 5.0, 7.0, 9.0, and 12 (*C* = 100 µM, *T* = 20 °C, *l* = 1.0 mm). (e) Proteostat aggregation assay on each monomers at the different pHs (*C* = 100 µM). Data are presented as ± SD, *n* = 4.

**Figure 6:**
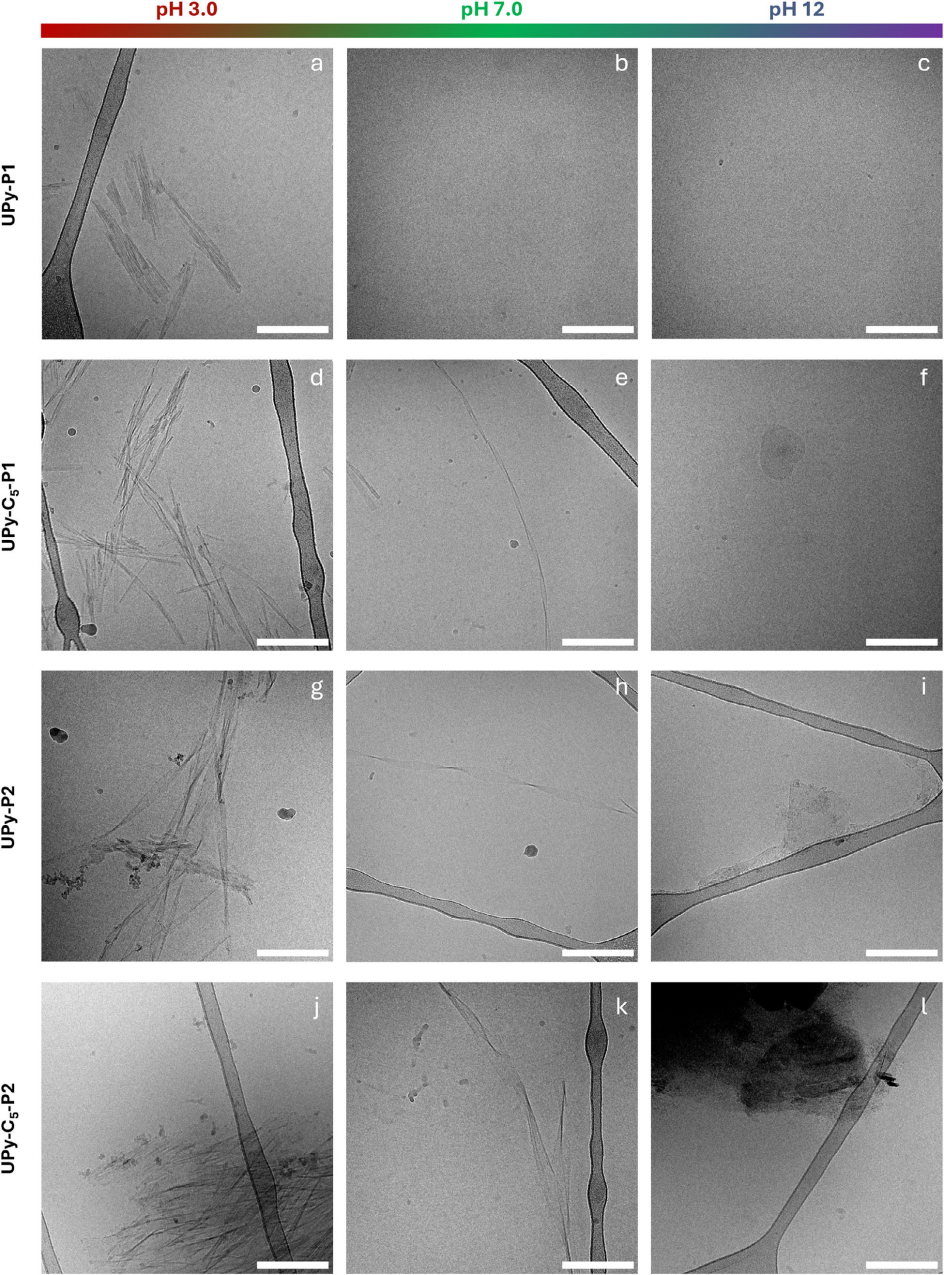
Representative cryoTEM images of (a–c) UPy‐P1, (d–f) UPy‐C_5_‐P1, (g–i) UPy‐P2 and (j–l) UPy‐C_5_‐P2 in water at nominal pH of 3.0 (left column), 7.0 (middle column), and 12 (right column) (*C* = 500 µM). All the micrographs are acquired at a nominal magnification of 24kx. Scale bars = 200 nm.

The thermal stability of the assemblies formed by each monomer is investigated through variable temperature (VT) CD (Figures S11 and 12) and VT‐UV–vis (Figures S13 and 14) spectroscopy. As expected, a decrease in the asymmetry of the supramolecular aggregates formed by the monomers is observed for each monomer upon heating the samples up to 80 °C, which is enhanced when the C_5_ spacer is present. However, the VT‐UV spectra of each monomer in solution show minor changes in the absorbance at 280 nm, suggesting great stability of the respective UPy tautomer and negligible tautomerization over the temperature range. Both the UPy unit^[^ ^48^, ^49^ ^]^ and the peptide sequences used in this study^[^ ^18^, ^50^, ^51^ ^]^ are pH responsive, due to enolate formation under basic conditions for the former and to the ionization of the carboxylic groups of the glutamic acid residues above pH = 5.0 for the latter.^[^ ^72^ ^]^ Therefore, the influence of the pH in the assembly process of each monomer is investigated at acidic, neutral and basic pH. The UV–vis spectra of each monomer at the different pHs (Figure S15) indicate the precipitation of all the monomers except for **UPy‐P1** at pH = 3.0, as demonstrated by the lack of UV–vis signal. This might be due to further aggregation resulting from the full protonation of the glutamic acid side chains.^[^ ^73^, ^74^ ^]^ Conversely, while negligible differences in absorbance are observed between pH = 5.0 and 9.0, enolate formation occurs only at pH = 12, as demonstrated by the appearance of the absorption maximum at 270 nm.^[^ ^75^ ^]^ The CD spectra of **UPy‐P1** (Figure 5a) exhibit no significant differences across varying pHs, except for a blue shift in the positive Cotton effect from 220 nm to 215 nm at pH = 12. In contrast, for all the other monomers, precipitation is confirmed at pH = 3.0 by the lack of signal, whereas a clear transition to random coils is observed only at pH = 12 upon enolate formation, irrespective of the peptide sequence involved. This is evidenced by the random coil signature present in the CD spectra of all the monomers at the most basic pH. Minor variations in the CD spectra of **UPy‐C_5_‐P1** (Figure 5b) and **UPy‐C_5_‐P2** (Figure 5d) are observed at pH = 5.0, 7.0, and 9.0, indicating comparable stability of the secondary structures formed by the two monomers in solution within the pH range. This may be due to a shift in the p*K*a of the glutamic acid side chains caused by assembly formation, leading to altered ionization within that pH range, as previously reported.^[^ ^76^, ^77^, ^78^ ^]^ Conversely, the CD signal intensity at 202 nm for **UPy‐P2** (Figure 5c) varies across the three pH conditions. At pH 7.0, the maximum CD signal is observed, while at pH 5.0, the intensity is slightly reduced, likely due to limited solubility of the monomer in acidic conditions. Nevertheless, other molecular rearrangements due protonation of the glutamic acid side chains cannot be ruled out. At pH 9.0, the signal decreases further, suggesting partial conversion of the β‐sheets into random coils, likely due to partial deprotonation of the glutamic acid side chains. This indicates a higher pH sensitivity for **UPy‐P2** compared to the more hydrophobic **UPy‐C_5_‐P2**. The Proteostat assay (Figure 5e) corroborates the aforementioned results. The fluorescence intensity reaches maximum values at pH 3.0 for **UPy‐C_5_‐P1**, **UPy‐P2**, and **UPy‐C_5_‐P2**, while remaining steady between pH 5.0 and 9.0 and dropping at pH 12 due to random coils formation. On the other hand, no significant differences in fluorescence intensity for **UPy‐P1** are observed over the pH range, in agreement with the CD measurements. Thus, these results indicate that the formed β‐sheets exhibit structural stability between pH = 5.0 and 9.0, whereas transition to random coils in solution occurs only at pH = 12, upon enolate formation on the UPy ring. The influence of pH on the morphology of the assemblies is then investigated through cryoTEM at pH = 3.0, 7.0, and 12. As expected, no visible assemblies are present in the micrographs of **UPy‐P1** at pH = 7.0 (Figure 6b) and 12 (Figure 6c). However, bundled structures are observed at acidic pH (Figure 6a), despite no difference with the other pHs is observed with the spectroscopic measurements. Therefore, the presence of aggregates at pH 3.0 might influenced by the five‐fold higher concentration used for the imaging. For the rest of the monomers, the acidic pH (Figure 6d, g, and j) induces bundling of the nanostructures observed at neutral pH (Figure 6e, h, and k), while the transition to more disordered structures, which might result from the aggregation of smaller random coils at a five‐fold higher concentration, occurs under basic conditions (Figure 6f, i and l), in agreement with the spectroscopic studies reported above. These results indicate that the acidic pH enhances the aggregation of each monomer in solution while the pH above 12 provides the transition to more disordered structures featuring the random coil conformation.

## 3 Conclusion

Four novel UPy self‐assembly monomers have been successfully synthesized featuring two distinct β‐sheet peptide sequences. The different peptide sequences have been shown to promote the formation of different intermolecular interactions and to affect the dimerization as well as the stacking of the UPy motifs. P1 leads to the formation of random coils and to antiparallel β‐sheets when combined with the alkyl spacer, while P2 results in parallel β‐sheets formation, with enhanced twisting upon incorporation of the C_5_ spacer. In addition, P1 has been shown to facilitate the dimerization of the UPy motif more than P2, probably as a consequence of a decreased steric hindrance provided by the alanine side chains compared to that of the valines in P2 and a potentially more favorable β‐sheets orientation. The introduction of an alkyl spacer between the UPy motif and the peptide sequence enhances both the assembly and the UPy dimerization, regardless of the peptide sequence used. However, while the alkyl spacer significantly decreases the mobility of both the UPy unit and the peptide sequence for UPy‐P1 and UPy‐C_5_‐P1, it slightly decreases the mobility of those moiety in UPy‐P2 and UPy‐C_5_‐P2. These findings suggest that the assembly of the monomers featuring the P2 sequence is mainly driven by the peptide sequence. Although the UPy dimerization decreases from P1 to P2, the UPy unit has been demonstrated to be responsible for the pH responsiveness of the assemblies formed by each monomer, providing the transition to random coils only at pH 12, upon enolate formation. On the other hand, all the assemblies showed structural stability in the pH range between 5.0 and 9.0, while at pH = 3.0 a significant increase in the assembly of all the monomers has been observed, probably due to the stabilization of the keto form of the UPy ring and to further aggregation provided by the protonation of all the carboxylic groups. This topic aids the design of UPy‐based molecules in combination with a variety of peptide sequences to develop novel materials with tailored responsivity and assembly properties.

## Supporting Information

Additional information is available free of charge, including detailed synthetic procedures, LC–MS characterization of the molecules, ^1^H‐NMR spectra, additional CD, UV–vis spectra, and SAXS analysis. The authors have cited additional references within the Supporting Information.^[^ ^79^, ^80^, ^81^, ^82^, ^83^ ^]^

## Conflict of Interests

The authors declare no conflict of interest

## Supporting information



Supporting Information

## Data Availability

The data that support the findings of this study are available from the corresponding author upon reasonable request.
