# Supplementary material for: Hybrid pH Responsive Supramolecular Polymers Through the Combination of the Ureido–Pyrimidinone Motif with β‐Sheet Peptide Sequences
Source: Chemistry. 2025 May 15;31(33):e202500429. doi: 10.1002/chem.202500429 (PMC12160978; doi:10.1002/chem.202500429)
Supplement: Supplementary file 1 — Supporting Information [file CHEM-31-e202500429-s001.docx]

**Supporting information**

**Table of Contents**

[1 Experimental Section S1](#_Toc183544713)

[**1.1 Materials and Methods** S1](#_Toc183544714)

[**1.2 Synthetic procedures** S4](#_Toc183544715)

[2 Molecular characterization S5](#_Toc183544716)

[3 ^1^H-NMR spectroscopy S7](#_Toc183544717)

[4 CD spectra S10](#_Toc183544718)

[5 UV-Vis spectra S12](#_Toc183544719)

[6 SAXS analysis S14](#_Toc183544720)

[7 References S15](#_Toc183544721)

# Experimental Section

## **Materials and Methods**

**Instrumentation:** All solvents were of AR quality and purchased from Biosolve. Deuterated compounds were obtained from Cambridge Isotope Laboratories and stored over 4 Å molecular sieves. Dry solvents were obtained using MBraun solvent purification system (MB SPS-800). Water for aqueous samples was purified on an EMD Millipore Milli-Q (MQ) Integral Water Purification System. Glassware was dried in an oven at 135 °C overnight prior to reactions under dry conditions. All solid state FT-IR spectra were recorded on a Perkin Elmer Spectrum Two FT-IR. All the samples were measured at room temperature from 500 cm^−1^ to 4000 cm^−1^ and were averaged over 128 scans. Liquid chromatography mass spectroscopy (LC-MS) spectra were acquired using a device consisting of multiple components: Shimadzu SCL-10 A VP system controller with Shimadzu LC-10AD VP liquid chromatography pumps (with an Alltima C18 3 u (50 × 2.1 mm) reversed-phase column and gradients of water), a Shimadzu DGU 20A3 prominence degasser, a Thermo Finnigan surveyor auto sampler, a Thermo Finnigan surveyor PDA detector and a Thermo Scientific LCW Fleet. All samples were dissolved in 1:1 H_2_O:MeCN in *ca*. 0.1 mg·mL^−1^ concentration.

**General sample preparation protocol:** Samples were prepared by dissolving the UPy molecules as solids in hexafluoro isopropanol (HFIP) at 10 mM. Aliquots of the stock solutions were diluted in MQ water at the proper concentration and freeze-dried overnight. Next, the freeze-dried powders were dissolved in MQ water at the desired pH and concentration. The resulting samples were then equilibrated overnight while shaking (600 rpm and 20 °C). In case of concentration dependent experiments, a stock solution at the highest concentration employed in the experiment was prepared as described above and diluted at different concentrations in the proper aqueous solvent. Next, the diluted samples were further sonicated for one hour and equilibrated overnight under shaking (600 rpm at 20 °C).

**Fluorescence Spectroscopy:** To record the fluorescence intensity, a MC SPARK 20M microplate reader by Tecan Group Ltd was used. The samples were measured in a Greiner 384 flat black well plate and data was processed in OriginPro 2019b by OriginLab.

**^1^H-NMR spectroscopy:** ^1^H-NMR experiments were carried out by heating the samples at 298 K using a Varian Unity Inova 500 MHz Spectrometer equipped with a 5 mm AutoX DB probe. Samples were prepared at 500 µM in D_2_O and equilibrated overnight at room temperature. Anhydrous dimethyl sulfoxide (DMSO) was diluted in D_2_O at 100 mM and further diluted into solutions to 50 µM as mobile internal standard for kinetic analysis. As a result, each sample contains 0.1 % v/v of the diluted DMSO solution (100 mM in D_2_O) which is expected not to interfere with the assembly process. Every spectrum was acquired over 128 scansions with relaxation delay of 10 sec. Quantification of the molar fraction (X) of each molecular moiety (namely: UPy, *β*-sheet sequence and glutamic acid side chains) was obtained from the integration of the respective peaks in the NMR spectrum of each molecule, using DMSO as internal standard (6H integrated as 600). Being the molar fraction corresponding to the glutamic acid side chains always exceeding 100 mol % due to limited accuracy of manual integration performed in MNova, the molar fraction (X) of each molecular moiety was normalized against the molar fraction of the glutamic acid side chains of each monomer.

**Circular Dichroism (CD) measurements:** The CD spectra were recorded on a J-815 JASCO spectrometer equipped with a JASCO Peltier PFD-425S/15 with a temperature range of 263 K to
383 K using the following settings; sensitivity: Standard, D.I.T: 0.5 sec, bandwidth: 1.0 nm, scanning speed: 50nm min^1^, data pitch: 0.5 nm in the range of 190 - 320 nm with a 1.0 mm path length quartz cell. An accumulation of three measurements was used and averaged to give the output spectra. Variable temperature CD (VT-CD) experiments were performed with the aforementioned settings and a cooling rate of 1.0 °C·min^-1^, data pitch: 1.0 °C and temperature interval: 10 °C The cooling curves monitored at 196 nm for **UPy-P1**, 202 nm for **UPy-P2**, 210 nm for **UPy-C_5_-P1** and 213 nm for
**UPy-C_5_-P2** were obtained by controlled cooling from 80 °C to 10 °C at a rate of 0.1 °C·min^-1^. Measurements were performed after heating the sample at 80 °C for 1 hour.

**UV-Vis measurements:** Variable temperature UV (VT-UV) measurements were carried out by heating the sample from 10 °C to 80 °C at a rate of 1.0 °C·min^-1^ using a JASCO V-750-UV-vis spectrometer equipped with a JASCO Peltier ETCR-762 with a temperature range of 263 K to 383 K and external circulating thermostat JASCO CTU-100 using the following parameters; response time: 0.24 sec, bandwidth: 1.0 nm, heating rate: 1.0 °C·min^-1^, data pitch: 1.0 °C, temperature interval:
5.0 °C. All spectroscopic measurements were performed using sealed Hellma quartz cuvettes with an optical path length of 1.0 mm and 9.0 mm metal spacer as heat bridge. All measurements were baseline corrected and averaged over three measurements. The samples were placed in the sample holder at 10 °C for 1 hour before heating to 80 °C to ensure comparable thermal history. The cooling curves monitored at 280 nm obtained were obtained by controlled cooling from 80 °C to 10 °C at a rate of 0.1 °C·min^-1^. Measurements were performed after heating the sample at 80 °C for 1 hour.

**Proteostat Assay:** The Proteostat Aggregation Assay Kit was purchased from *Enzo Life Science, Inc.* All the materials were weighed in a clean glass vial and dissolved in MQ water (pH 7.4) by sonication for one hour to a final concentration of 300 µM. These stock solutions were further diluted to 1, 2, 5, 10, 25, 50, 75, 100, 150, 200 and 300 µM in MQ water (pH 7.4) and sonicated for one hour. Next, the Proteostat solution was prepared by mixing 1.092 µL of Proteostat stock solution in 206.81 µL of MQ water and 2.1 µL of assay buffer. 1 µL of this solution was added to 9 µL of each peptide solution in a Greiner 384 flat black weelplate. The solutions were incubated for 30 minutes while shaking at 550 rpm at 20 °C. The fluorescence intensity was measured on a MC SPARK 20M microplate reader by Tecan Group Ltd. The samples were measured in a Greiner 384 flat black well plate, using an excitation wavelength of 550 nm and an emission wavelength of 600 nm and a band width of 20 nm. Samples were shaken for 2 s before every measurement and the experiment was done four times. Data was processed in OriginPro 2019b by OriginLab. For kinetics analysis, each monomer was molecularly dissolved in HFIP to 20 mM and diluted to 100 µM in a solution containing the fluorescence probe prepared as described above. The fluorescence intensity at 600 nm was then recorded every ten minutes for sixteen hours. The fluorescence intensity at 600 nm of the blank with only MQ water and the Proteostat solution is subtracted from the fluorescence intensity at 600 nm of each sample. The resulting signals are presented as ± SD and plotted against the concentration or time.

**Cryo-TEM:** Vitrified thin films for cryoTEM analysis were prepared using an automated vitrification robot (FEI Vitrobot Mark IV) by plunge vitrification in liquid ethane. Before vitrification, a 200-mesh copper grid covered either with a Lacey carbon film (Electron Microscopy Sciences) or with a Quantifoil R 2/2 holey carbon film (Quantifoil Micro Tools GmbH) was surface plasma treated for 40seconds using a Cressington 208 carbon coater. CryoTEM imaging was carried out on the Glacios (Thermo Fisher), equipped with a field emission gun (X-FEG), Ceta 16M camera and a Falcon 4i direct electron detector. The microscope was operated at 200kV acceleration voltage in bright-field TEM at a nominal magnification of 6.500× and a dose rate of 2 e^-^/Å^2^·s; or at 24.000× magnification and a dose rate of 4 e^-^/Å^2^·s; both with a 1s image acquisition time.

**Small Angle X-ray scattering (SAXS):** SAXS measurements were carried out using a SAXSLAB GANESHA system with a two-dimensional Pilatus 300 K detector, using Microfocus Cu source, Xenocs Genix 3D. The samples were loaded into 2 mm quartz capillaries and the scattering intensity *I(q)* was recorded at a q-range of 0.007 < q < 0.212 Å^−1^. The 2D SAXS patterns were azimuthally averaged to produce one-dimensional intensity profiles, *I(q) vs. q*, using the two-dimensional data reduction program SAXSGUI. The scattering spectra of the solvent were subtracted from the corresponding solution data using the Irena package in Igor Pro 9 from WaveMetrics (Portland, Oregon).^[1]^

The scattering pattern of **UPy-P1** was fitted to the Debye Gaussian coil model:^[2]^

| (S1) | $I\left( q \right)=2\cdot I_{0}\cdot\frac{\left\{ \exp\left[ -\left( qR_{g} \right)^{2} \right]+\left( qR_{g} \right)^{2}-1 \right\}}{\left( qR_{g} \right)^{4}}$ |
| --- | --- |

Where *q* is the scattering vector and *R_g_* is the radius of gyration.

The scattering patterns of **UPy-C_5_-P1**, **UPy-P2** and **UPy-C_5_-P2** were fitted to the parallelepiped model:^[3,4]^

| (S2) | $I\left( q \right)=\frac{\left( \Delta\rho V \right)^{2}}{V}\cdot\left[ {\frac{\sin\left( \frac{1}{2}qA \right)}{\left( \frac{1}{2}qA \right)}}^{2} \right]\cdot\left[ {\frac{\sin\left( \frac{1}{2}qB \right)}{\left( \frac{1}{2}qB \right)}}^{2} \right]\cdot\left[ {\frac{\sin\left( \frac{1}{2}qC \right)}{\left( \frac{1}{2}qC \right)}}^{2} \right]$ |
| --- | --- |

Where $\Delta\rho=\left( \rho_{p}-\rho_{sol} \right)$ is defined as the contrast, $V=ABC$ is the volume $(A<B<C)$.

## **Synthetic procedures**

**N-(6-methyl-4-oxo-1,4-dihydropyrimidin-2-yl)-1H-imidazole-1-carboxamide (UPy-CDI)**

Scheme S1. Synthesis of UPy-CDI.

A round bottom flask was charged with **1** (313 mg; 2.5 mmol; 1.0 eq.), 1, 1'-carbonyldiimidazole (CDI) (811 mg; 5.0 mmol; 2.0 eq.) and dimethyl sulfoxide (DMSO) (4.0 mL). The resulting suspension was heated up to 80 °C and stirred for 24 hours under Ar atmosphere. After 24 hours, the mixture was cooled down to room temperature and acetone was added. The solid material was isolated through filtration, washed with acetone and dried under vacuum to yield the pure compound (548 mg, 2.4 mmol) as a white fluffy powder in quantitative yield. Due to the low solubility of the obtained compound, no characterization was performed and it was used directly into the next step without further purification.

**Synthesis of UPy-PAs and UPy-C_5_-PAs**

Scheme S2. Synthesis of UPy-P1,2 and UPy-C_5_-P1,2.

Peptides were synthesized using the Fmoc solid phase peptide synthesis strategy by Merrifield,^[5]^ synthesizing the peptide from *C-* to *N*-terminus on a Fmoc-Glu*(*O*t*Bu*)-*Wang (100-200 mesh) resin which was swollen in DMF (7.0 mL) for 1 h before use. Fmoc group was removed before every coupling by two consecutive deprotection steps (2 × 10 min) with 20% v/v piperidine in DMF (10 mL) at room temperature. After deprotection, the resin was washed five times with DMF (7.0 mL). Amino-acid couplings were performed with Fmoc-protected amino-acid (4 equivalents), O-(1H-6-Chlorobenzotriazole-1-yl)-1,1,3,3-tetramethyluronium hexafluorophosphate (HCTU) (4 equivalents) and *N,N*-diisopropylethylamine (DIPEA) (16 equivalents) in DMF (6.0 mL) under shaking at room temperature for 10-20 minutes. The coupling was performed twice for every amino-acid. The C_5_ spacer of the **P1** and **P2** was coupled after Fmoc deprotection of the last amino acid at the *N-*terminus using 6-(Fmoc-amino)hexanoic acid (4 equivalents), HCTU (4 equivalents) and DIPEA (16 equivalents) in DMF (6.0 mL) under shaking at room temperature overnight. For the UPy coupling, the desired amount of peptide on the resin was treated with 20 % v/v piperidine in DMF (2 × 10 min), washed with DMF, dried with DCM and transferred to an oven dried round bottom flask equipped with a small stirring bar along with **UPy-CDI** (4 equivalents) and anhydrous THF (4.0 mL). The mixture was then carefully stirred at reflux under Argon atmosphere overnight. Next, the resin was filtered and washed with methanol (50 mL), water (50 mL), DMF (50 mL) and DCM (50 mL). The peptides were cleaved from the solid support by using 4.0 mL of a cleavage cocktail (95% TFA, 2.5% TIPS, 2.5% H_2_O) at room temperature for 4 hours. The suspension was then filtered and precipitated in cold Et_2_O (2 × 40 mL). The pellet was collected by centrifugation and dried by lyophilization from water. The peptides were purified by HPLC with the Atlantis T3 column (flowrate 20 mL/min). The gradient started with 5% MeCN, which was kept constant for 1 min, then the MeCN content was increased linearly to 100% within 20 min.

# Molecular characterization


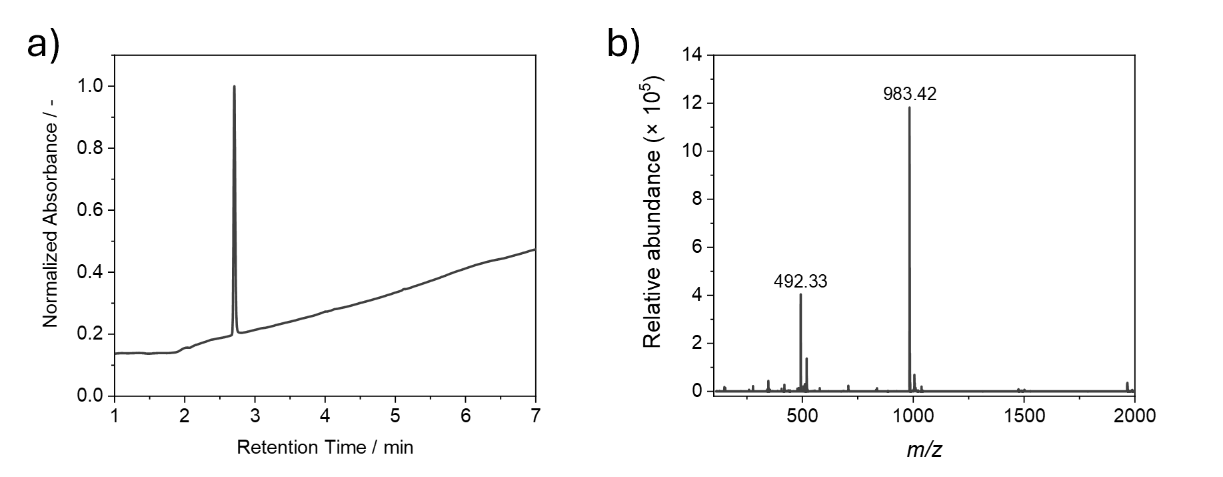


Figure S1. LC-MS (ESI+, H_2_O:MeCN = 1:1) spectrum of compound UPy-P1 (t_R_ = 2.71 min; calculated m/z = 982.4; found m/z =
[M+H]^+^ = 983.42; [M+2H]^+^ = 492.33).


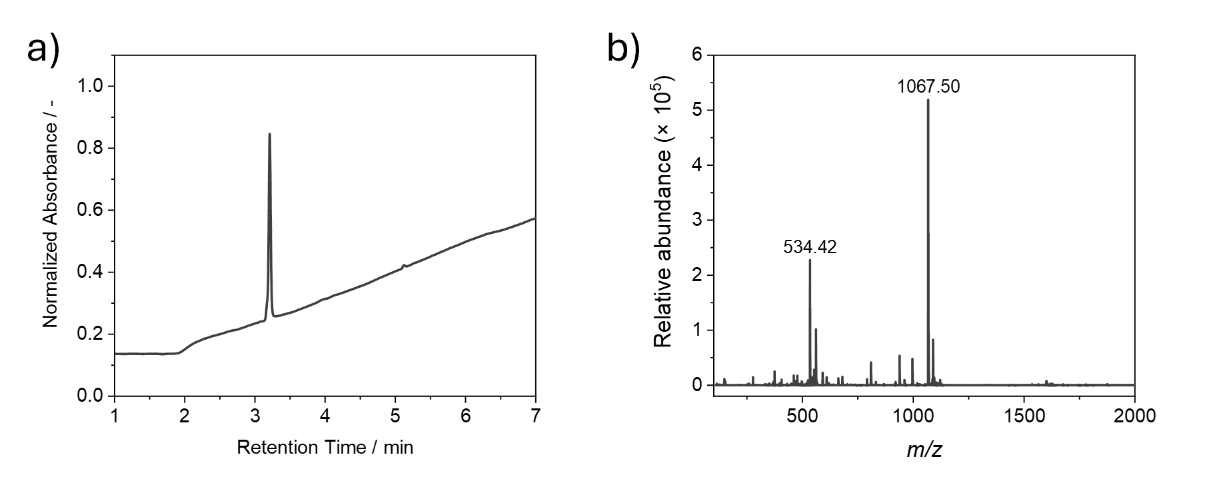


Figure S2. LC-MS (ESI+, H_2_O:MeCN = 1:1) spectrum of compound UPy-P2 (t_R_ = 3.21 min; calculated m/z = 1066.49; found m/z = [M+H]^+^ = 1067.5; [M+2H]^+^ = 534.42).


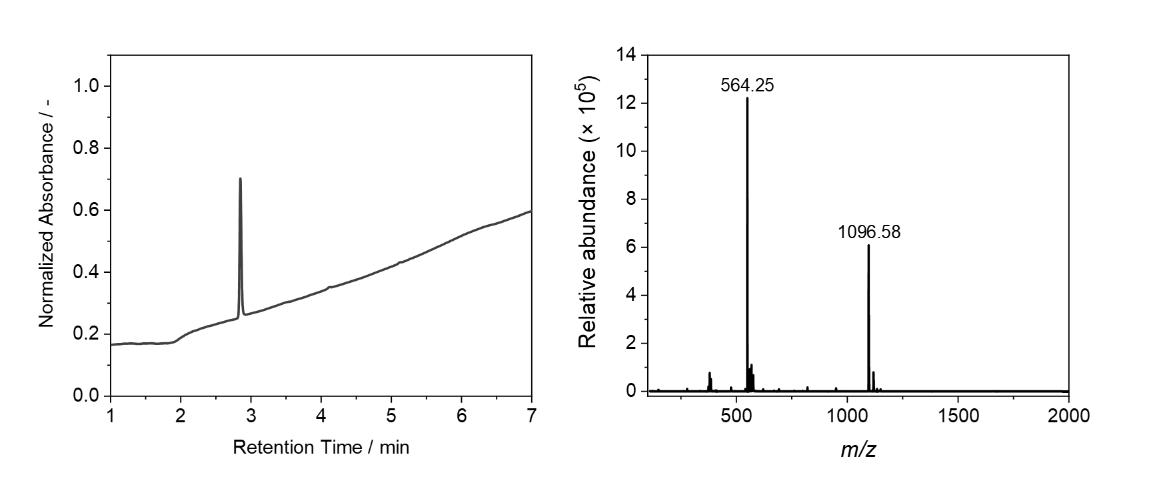


Figure S3. LC-MS (ESI+, H_2_O:MeCN = 1:1) spectrum of compound UPy-C_5_-P1 (t_R_ = 2.85 min; calculated m/z = 1095.48; found m/z = [M+H]^+^ = 1096.58; [M+2H]^+^ = 564.25).


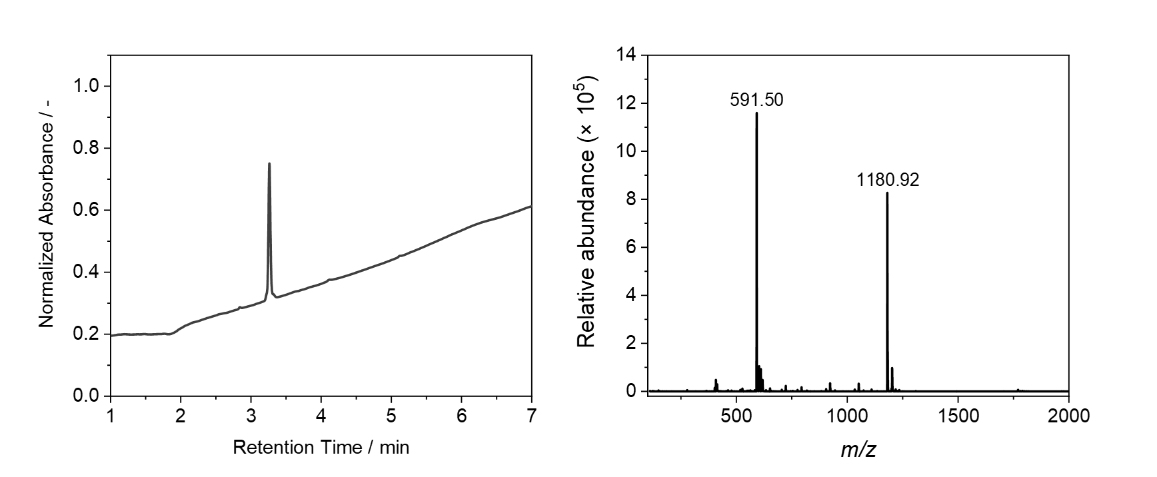


Figure S4. LC-MS (ESI+, H_2_O:MeCN = 1:1) spectrum of compound UPy-C_5_-P2 (t_R_ = 3.26 min; calculated m/z = 1179.58; found m/z = [M+H]^+^ = 1180.92; [M+2H]^+^ = 591.50).

# ^1^H-NMR spectroscopy


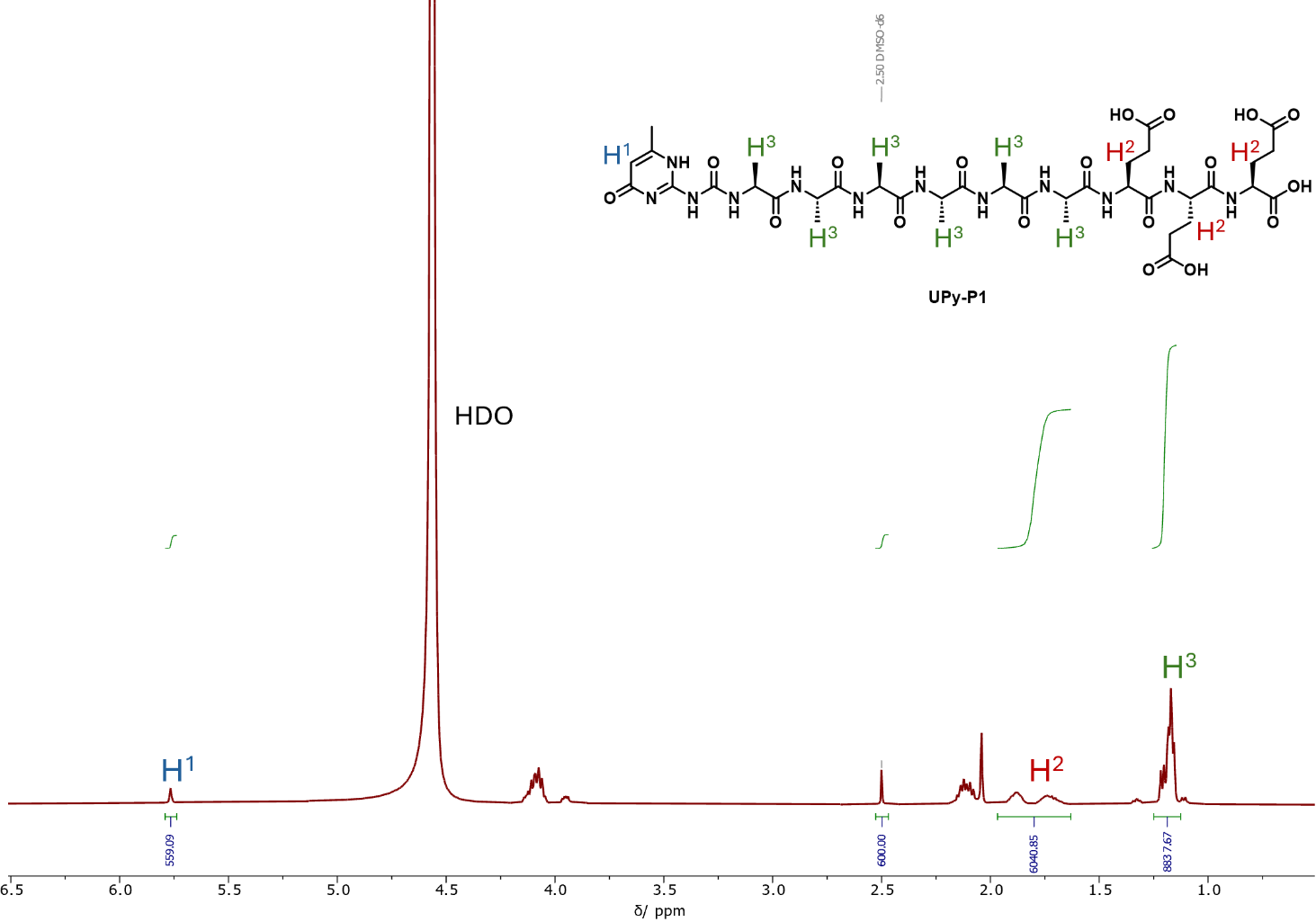


Figure S5. ^1^H-NMR of the assembled UPy-P1 in D_2_O (C = 500 µM) with DMSO as mobile internal standard (C = 50 µM), showing its mobile fraction.


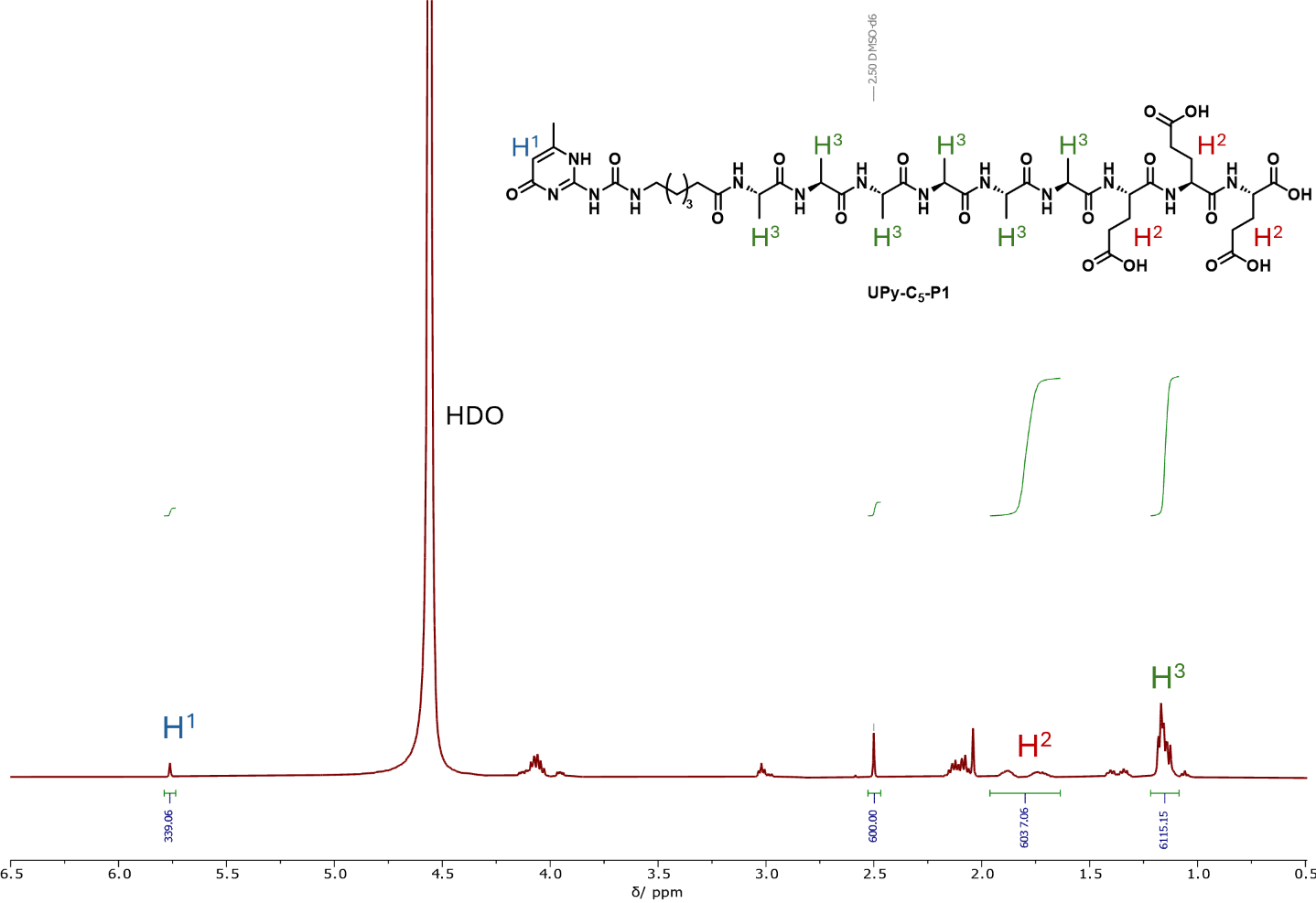


Figure S6. ^1^H-NMR of the assembled UPy-C_5_-P1 in D_2_O (C = 500 µM) with DMSO as mobile internal standard (C = 50 µM), showing its mobile fraction.


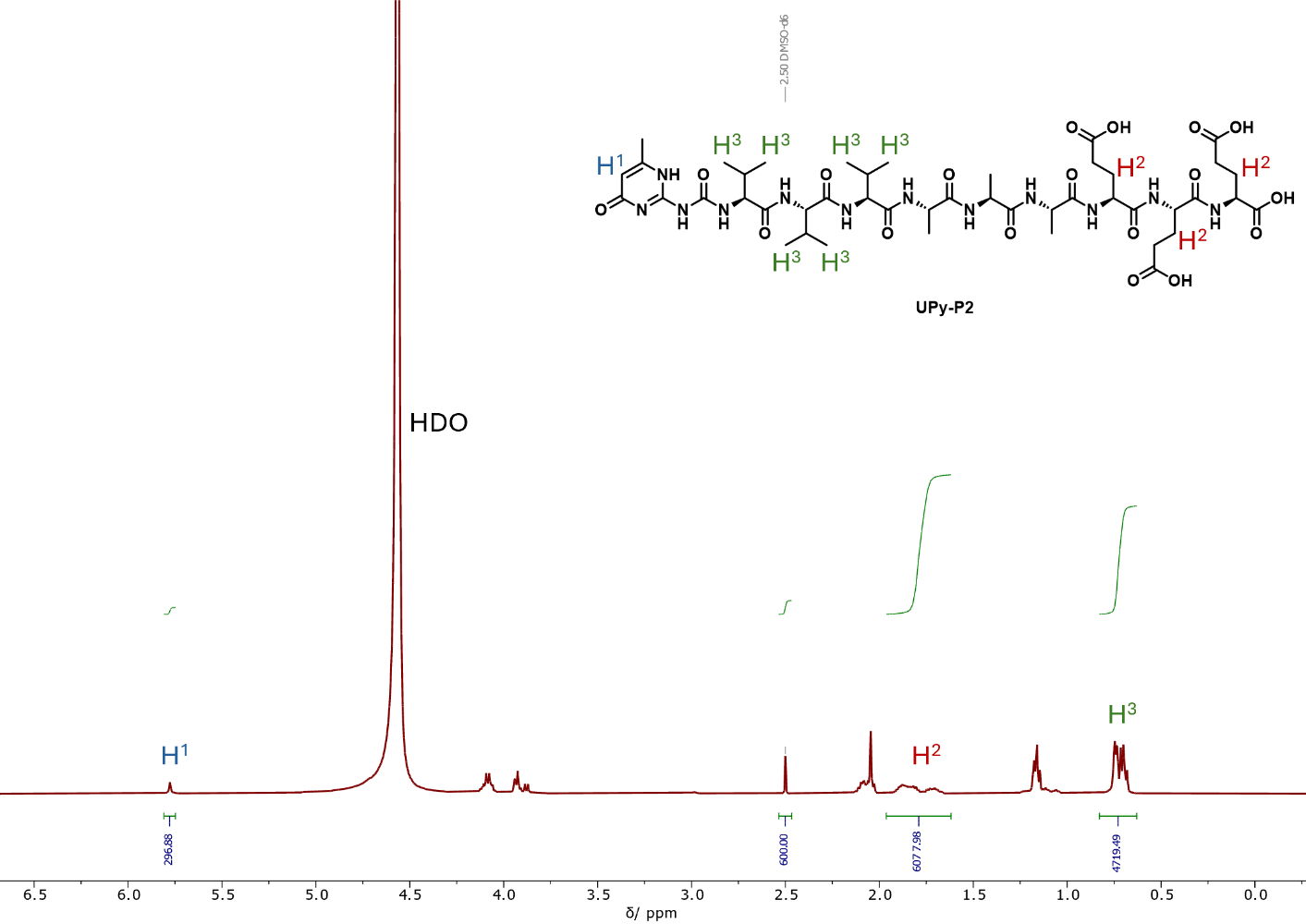


Figure S7. ^1^H-NMR of the assembled UPy-P2 in D_2_O (C = 500 µM) with DMSO as mobile internal standard (C = 50 µM), showing its mobile fraction.


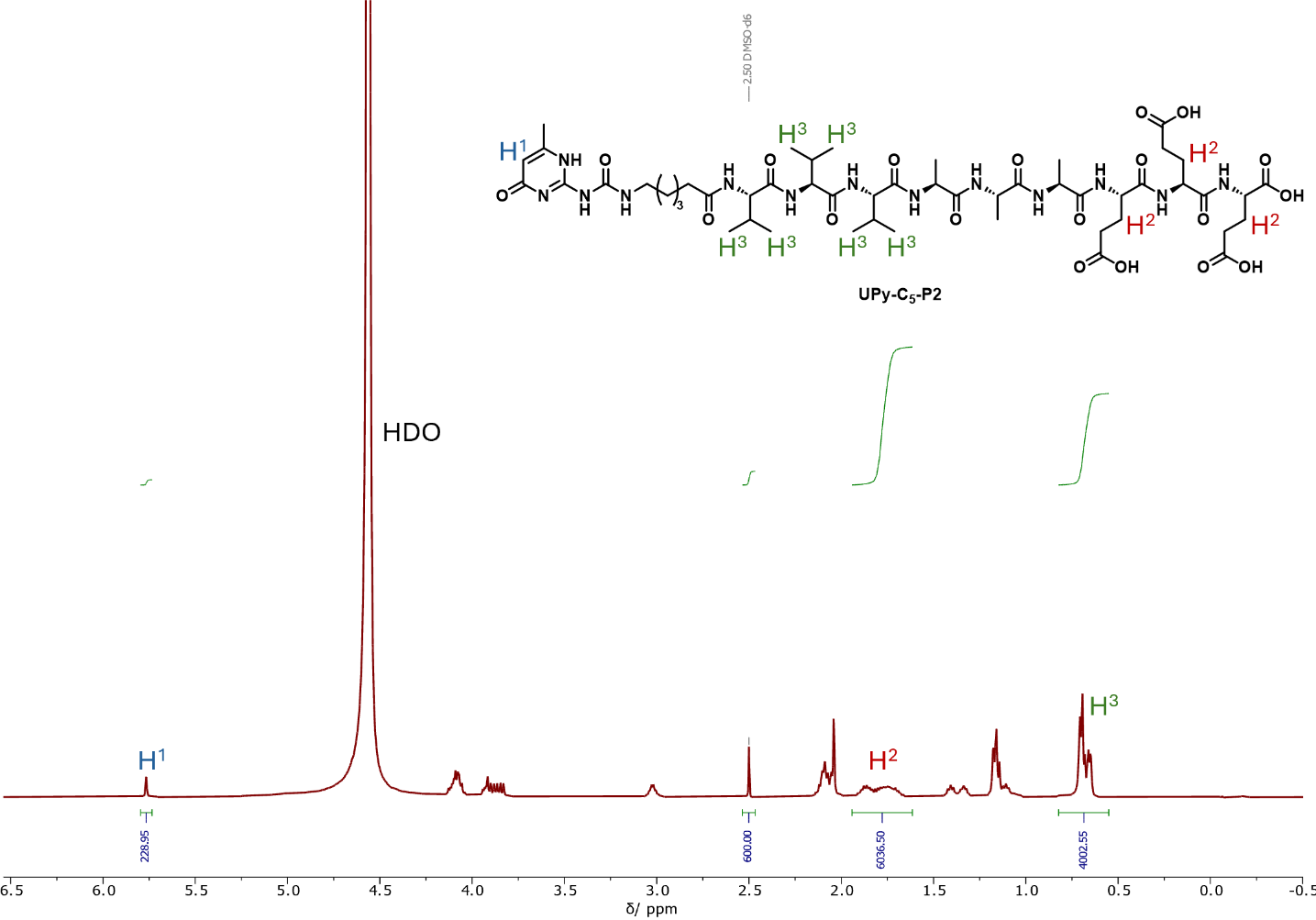


Figure S8. ^1^H-NMR of the assembled UPy-C_5_-P2 in D_2_O (C = 500 µM) with DMSO as mobile internal standard (C = 50 µM), showing its mobile fraction.


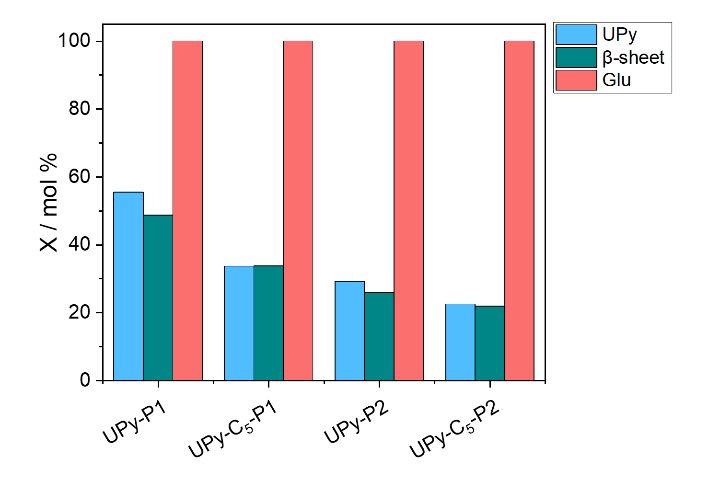


Figure S9. NMR quantification of the mobile visible fraction of UPy, β-sheet and glutamic acid side chains for each monomers (C = 500 µM in D_2_O) based on the integration of the respective peaks in the ^1^H-NMR spectra of the assembled monomers in D_2_O reported above.

# CD spectra


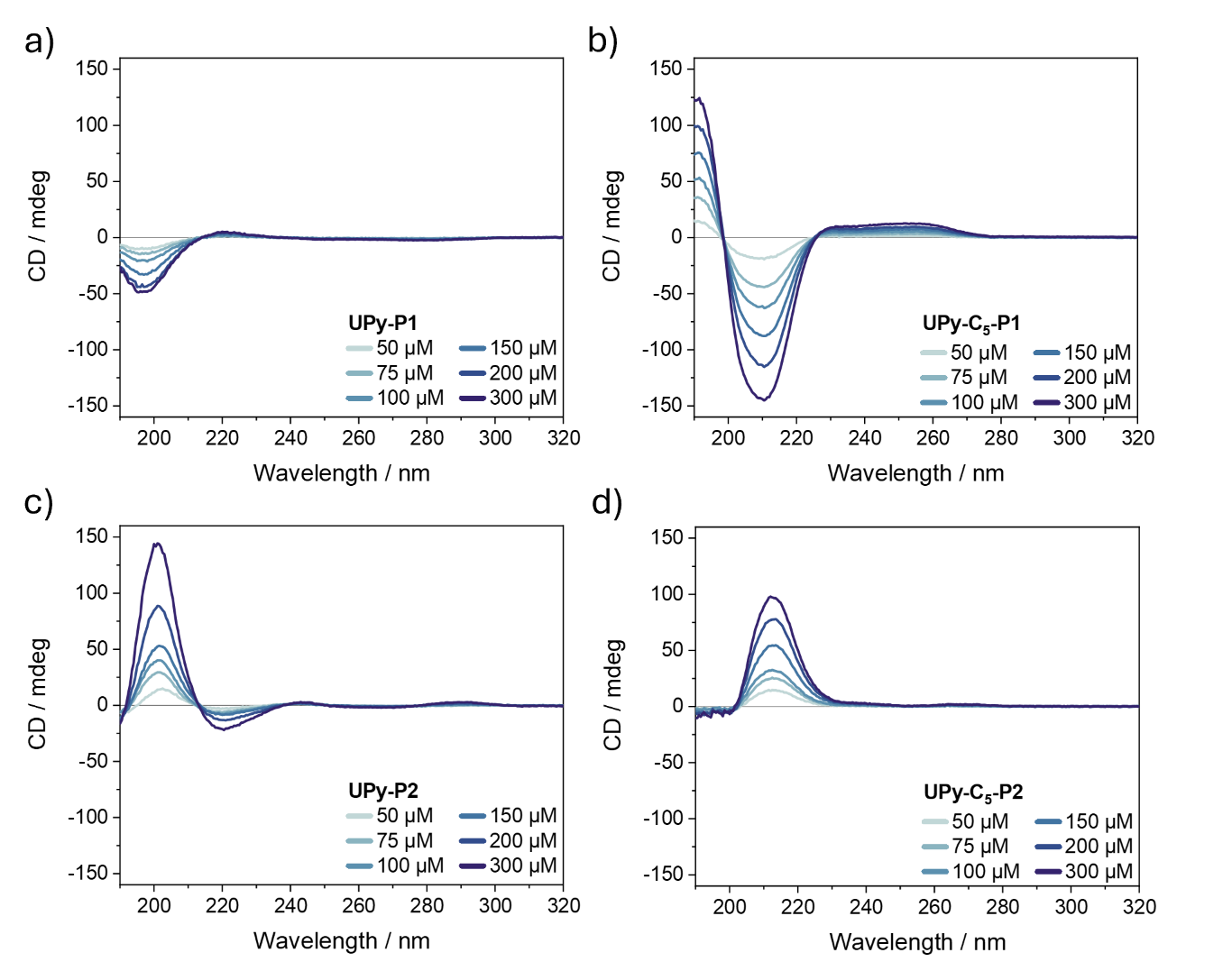


Figure S10. Concentration dependent CD spectra of (a) UPy-P1, (b) UPy-C_5_-P1, (c) UPy-P2 and (d) UPy-C_5_-P2 in MQ water at neutral pH (C = 50 µM to 300 µM, T = 20 °C, *l* = 1.0 mm).


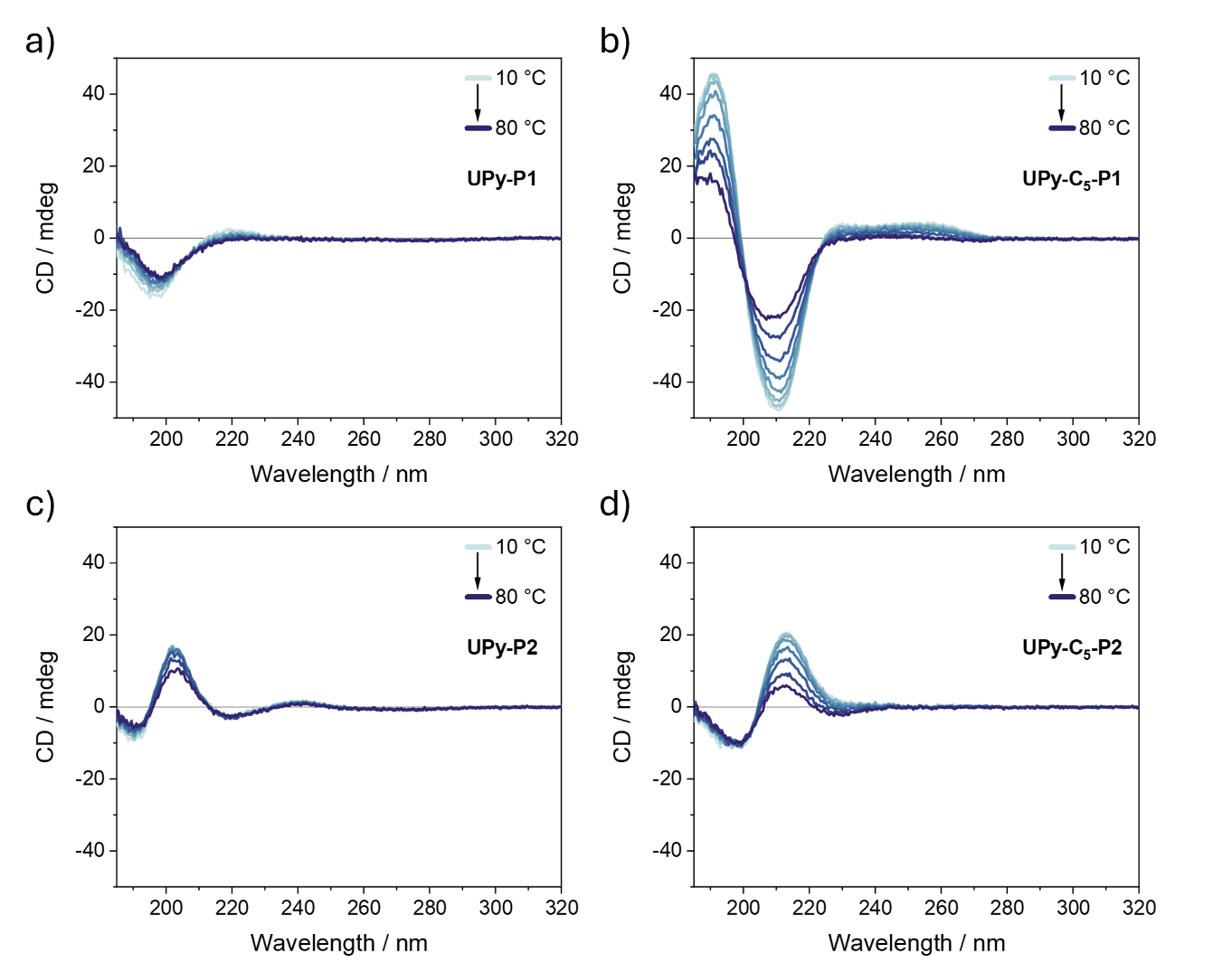


Figure S11. VT-CD spectra of (a) UPy-P1, (b) UPy-C_5_-P1, (c) UPy-P2 and (d) UPy-C_5_-P2 in MQ water at neutral pH (C = 100 µM, *l* = 1.0 mm, 1.0 °C min^-1^).


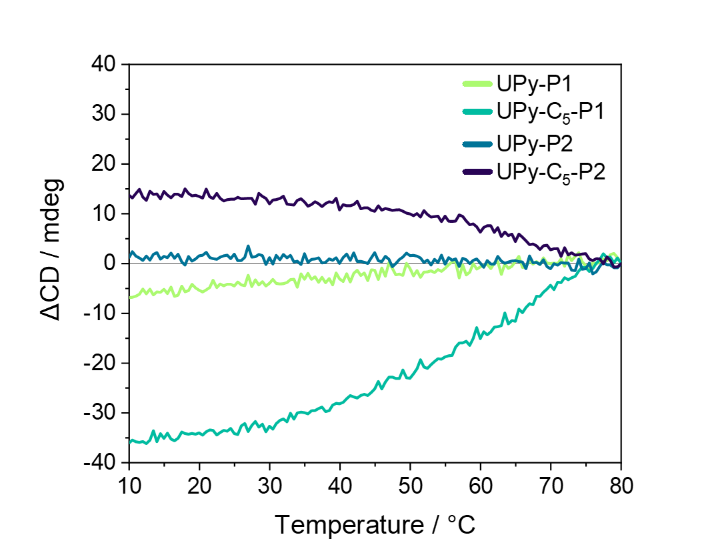


Figure S12. VT-CD cooling curves monitored at 196 nm for UPy-P1, 202 nm for UPy-P2, 210 nm for UPy-C_5_-P1 and 213 nm for
UPy-C_5_-P2 in MQ water at neutral pH (C = 100 µM, *l* = 1.0 mm, 0.1 °C min^-1^).

# UV-Vis spectra


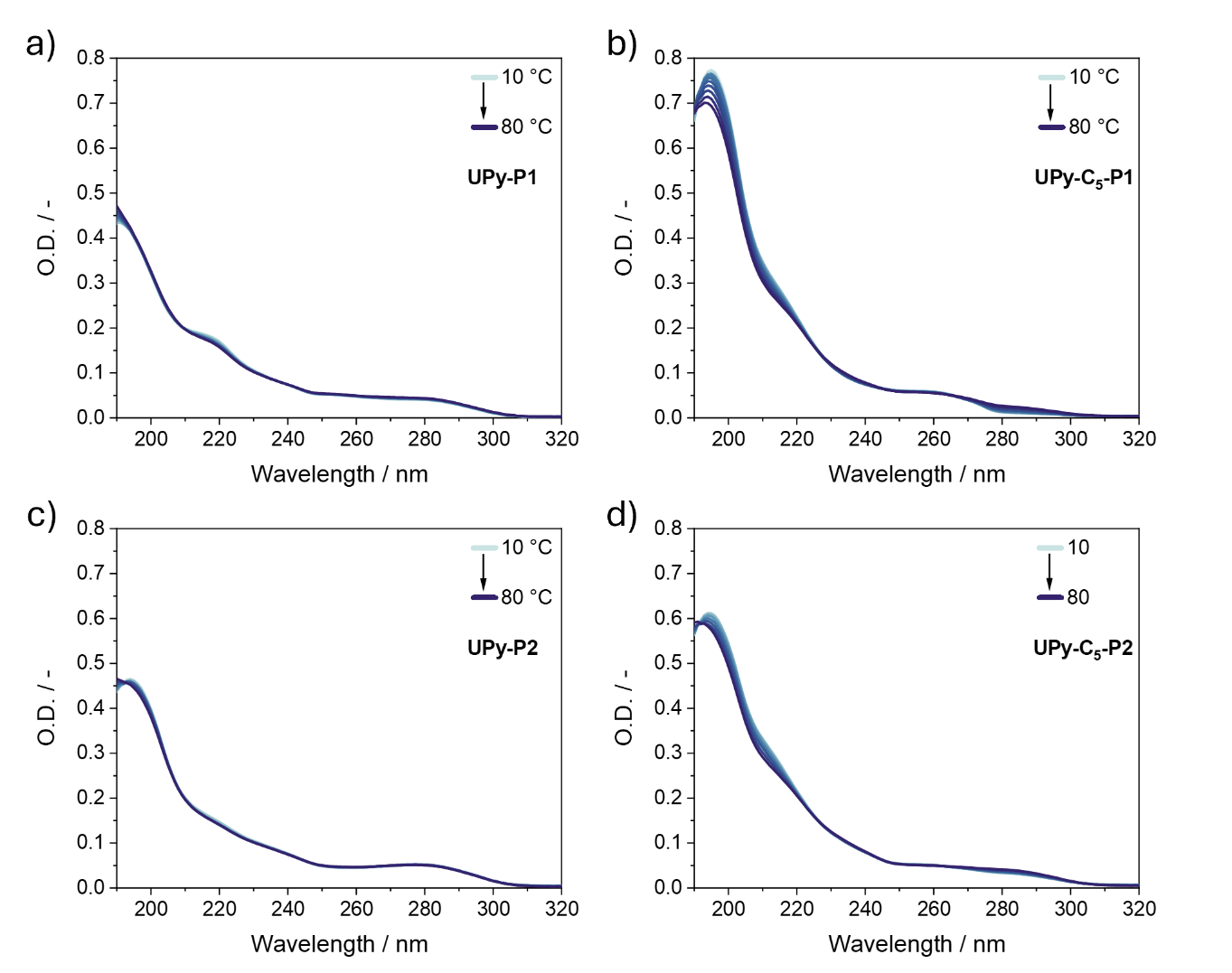


Figure S13. VT-UV spectrum of (a) UPy-P1, (b) UPy-C_5_-P1, (c) UPy-P2 and (d) UPy-C_5_-P2 in MQ water at neutral pH (C = 100 µM,
*l* = 1.0 mm, 1.0 °C min^-1^).


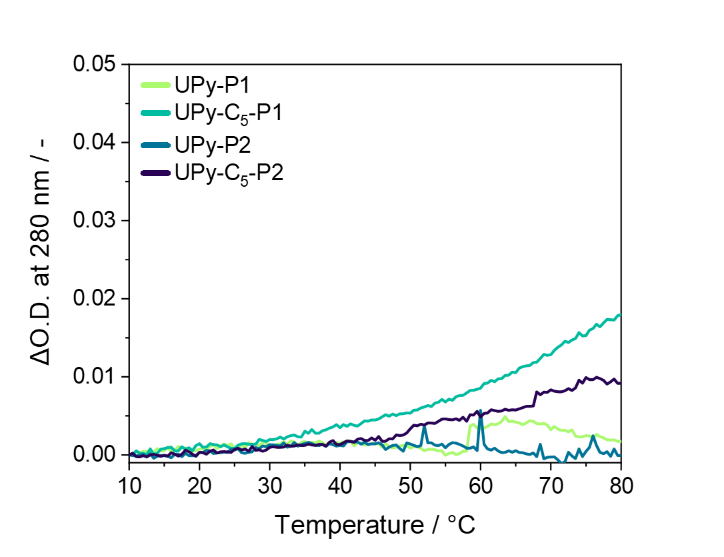


Figure S14. Cooling curves of the assembled monomers monitored at 280 nm in MQ water at neutral pH (C = 100 µM, *l* = 1.0 mm,
0.1 °C min^-1^).


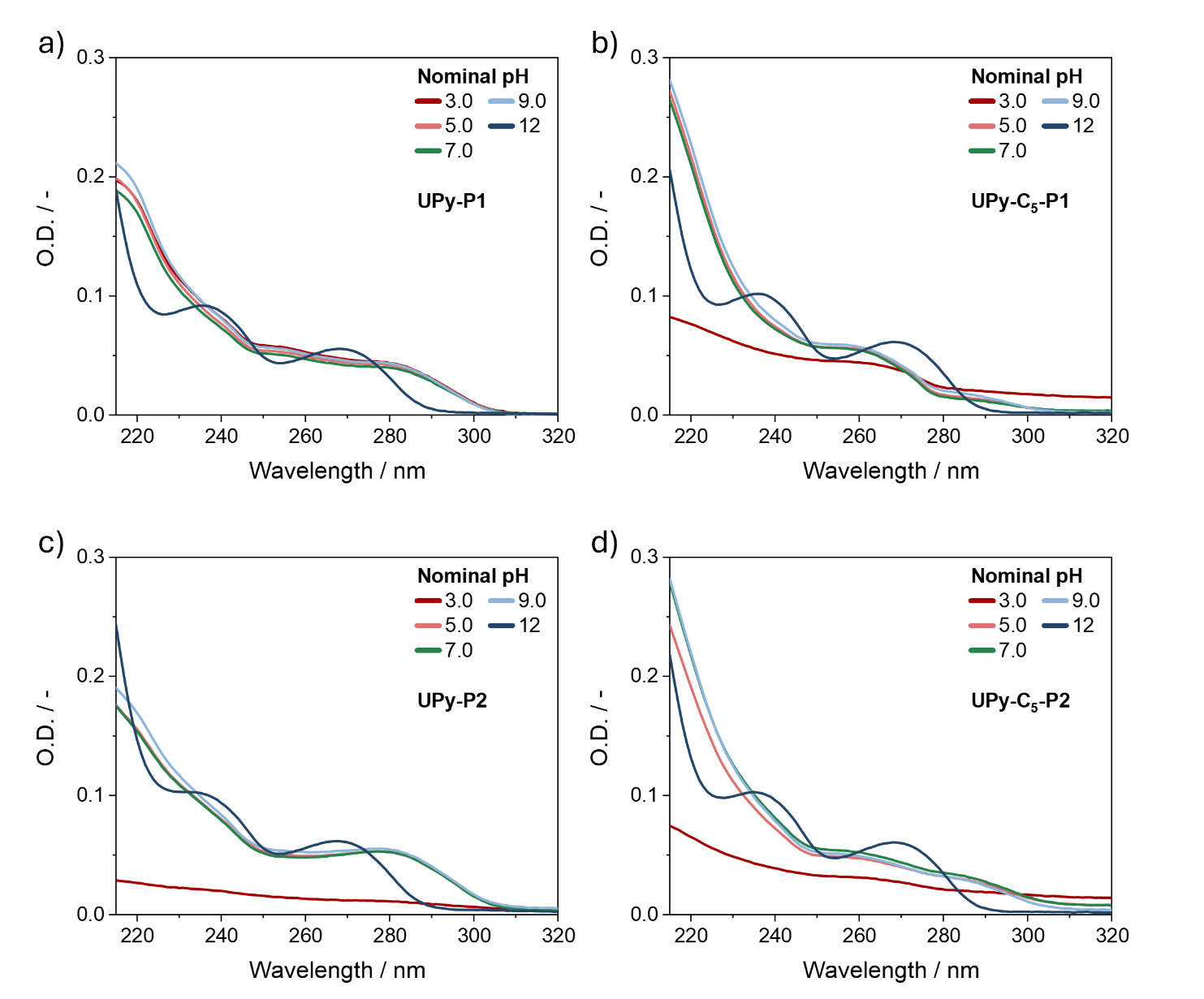


Figure S15. UV spectrum of (a) UPy-P1, (b) UPy-C_5_-P1, (c) UPy-P2 and (d) UPy-C_5_-P2 in MQ water (C = 100 µM, T = 20 °C, *l* = 1.0 mm) at pH 3.0, 5.0, 7.0, 9.0 and 12.

# SAXS analysis

Table S1. Summary of the parameters obtained from the fits of each sample in MQ water (C = 0.5 mM).

| **Molecule** | **UPy-P1** | **UPy-C5-P1** | **UPy-P2** | **UPy-C5-P2** |
| --- | --- | --- | --- | --- |
| Model | Mono Gauss Coil | Parallelpiped | Parallelpiped | Parallelpiped |
| *I_0_* | 94.863 | - | - | - |
| *R*_g_ / nm | 10.7 ± 0.3 | - | - | - |
| Length a / nm | - | 7.0 ± 0.1 | 4.8 ± 0.2 | 6.7 ± 0.1 |
| Length b / nm | - | 18.2 ± 0.2 | 16.2 ± 0.3 | 42.7 ± 1.3 |
| Length c / nm | - | 124.4 ± 13 | 543.2 ± 90 | 121.3 ± 8.5 |
| χ^2^ | 1.9405 | 1.7172 | 1.5628 | 1.5658 |

# References

[1] J. Ilavsky, P. R. Jemian, *J. Appl. Crystallogr.* **2009**, *42*, 347–353.

[2] P. Debye, *J. Phys. Chem.* **1947**, *51*, 18–32.

[3] R. Nayuk, K. Huber, *Zeitschrift für Phys. Chemie* **2012**, *226*, 837–854.

[4] P. Mittelbach, G. Porod, *Acta Phys. Austriaca* **1961**, *14*, 185–211.

[5] R. B. Merrifield, *J. Am. Chem. Soc.* **1963**, *85*, 2149–2154.
